# Current Insights into the Phytochemistry and Pharmacological Properties of *Ruscus aculeatus*

**DOI:** 10.3390/molecules30224417

**Published:** 2025-11-15

**Authors:** Wiktoria Pacuła, Ireneusz Sowa, Marcin Feldo, Filip Graczyk, Rafał Patryn, Magdalena Wójciak

**Affiliations:** 1 Department of Analytical Chemistry, Medical University of Lublin, Chodźki 4a, 20-093 Lublin, Poland; wiktoria.pacula@umlub.edu.pl (W.P.); ireneusz.sowa@umlub.edu.pl (I.S.); 2 Chair and Department of Vascular Surgery and Angiology, Medical University of Lublin, 11 Staszica St., 20-081 Lublin, Poland; martinf@interia.pl; 3 Department of Pharmaceutical Botany and Pharmacognosy, Ludwik Rydygier Collegium Medicum, Nicolaus Copernicus University, 9 Marie Curie-Skłodowska Street, 85-094 Bydgoszcz, Poland; filip.graczyk@cm.umk.pl; 4 Department of Humanities and Social Medicine, Medical University of Lublin, Chodźki 7, 20-093 Lublin, Poland; rafal.patryn@umlub.edu.pl

**Keywords:** chronic venous disorder, ruscogenin, butcher’s broom

## Abstract

*Ruscus aculeatus* L. (butcher’s broom), a member of the Asparagaceae family, is a perennial shrub native to the Mediterranean and Black Sea regions and naturalized in parts of Europe and North America. Traditionally, the rhizome and root of this species have been employed in folk medicine for the treatment of venous insufficiency, hemorrhoids, edema, and various dermatological and urinary ailments. These therapeutic applications are attributed primarily to the presence of steroidal saponins such as ruscogenin and neoruscogenin, as well as flavonoids and other bioactive compounds. In recent decades, *R. aculeatus* extracts have been incorporated into numerous pharmaceutical and cosmetic preparations, particularly those intended to improve venous tone, reduce swelling, and alleviate symptoms of chronic venous disorders. However, despite its widespread use, studies regarding *R. aculeatus* remain limited. Many investigations have focused on complex formulations such as Cyclo 3 Fort, which also contains hesperidin methylchalcone and ascorbic acid, making it difficult to attribute the observed effects solely to *R. aculeatus*. This review provides an overview of the current knowledge on the phytochemistry and pharmacological activities of *R. aculeatus*. The available data support the plant’s traditional use, yet further well-designed experimental and clinical studies are needed to clarify its mechanisms of action, confirm its therapeutic potential, and ensure safety and standardization in medicinal preparations.

## 1. Introduction

*Ruscus* L. is a genus of plants in the family Asparagaceae, which includes six species distributed from Macaronesia through Europe and North Africa [1]. Plants of this genus are primarily found in forests, often growing in deep shade. Among these species, *Ruscus aculeatus* L. is the most widespread. It occurs in forests on calcareous soils, in shrubland formations, shaded rocky areas, and moist habitats [2]. *R. aculeatus* occurs naturally in countries bordering the Mediterranean and Black Sea, as well as in southeastern Great Britain. The species has also been introduced to Mexico, Germany, and Ireland [3]. The scientific name of the genus derives from the ancient Latin word ruscum, referring to a thorny plant [4]. In English, *R. aculeatus* is commonly known as “butcher’s broom,” a name originating from the traditional use of its branches to clean butchers’ wooden blocks of blood and fat residues. Similarly, its Italian name (Pungitopo), its German name (Mausedorn)—both literally meaning “mouse sting”—and its Polish name (Myszopłoch kolczasty) refer to the custom of placing the plant’s shoots over food to keep rodents and other pests away [5].

The root and rhizome of *R. aculeatus* have traditionally been used in folk medicine for the treatment of venous insufficiency, hemorrhoids, and edema due to their vasoconstrictive, anti-inflammatory, and diuretic properties. Infusions and decoctions have also been employed to alleviate symptoms of colitis and diarrhea [6,7]. In Palestinian, South Balkan, and Eastern Mediterranean traditional medicine, *R. aculeatus* is mentioned as a remedy for various skin-related ailments [8,9]. In Turkey, decoctions prepared from the rhizomes are traditionally applied to relieve eczema [10], while infusions or decoctions from the aerial parts are used in Greece to treat itching and skin cracks [11]. Additionally, root and stem infusions are reported to be used as treatments for kidney stones and nephritis in Turkey [10,12]. In central Italy, topical applications of the plant have been used to manage warts and frostbite [6,13]. In addition, the species has been cultivated as an ornamental plant and as a food plant. Young shoots of *R. aculeatus* are eaten like asparagus in countries of the Istria region and Italy [14]. The seeds were also used in the past as a coffee substitute [5].

Despite its wide range of traditional uses, the most recognized property of *R. aculeatus* is its venoactive effect, which has found application in modern phytotherapy, particularly in the management of disorders associated with venous insufficiency. The rhizome is described in the EU herbal monograph as a remedy used to relieve leg discomfort and heaviness and to support the treatment of hemorrhoids [15]. Over recent decades, extracts of *R. aculeatus* have been incorporated into different preparations aimed at improving venous tone, reducing swelling, and alleviating symptoms of chronic venous disorders [16,17,18]. It has also been included as one of the components of a nanoemulsion tested in patients with advanced hemorrhoidal disease [19]. In medicine, the underground parts of the plant are used, as they are rich in active compounds such as steroidal saponins, which are believed to contribute to its therapeutic effects [5].

However, despite its widespread use, the number of studies evaluating its efficacy remains limited, and most research articles focus on Cyclo 3 Fort, which is actually a mixture of dry extract of butcher’s broom, hesperidin methylchalcone (HMC), and ascorbic acid (vitamin C) [20]. Therefore, the observed effects are difficult to attribute specifically to the activity of *R. aculeatus* itself.

Given the ongoing popularity of *R. aculeatus* in various formulations, there is a clear need to summarize the existing scientific data and identify gaps in current knowledge. This review aims to provide an up-to-date overview of the phytochemistry, pharmacological activities, and clinical applications of *R. aculeatus*, helping to improve the understanding of its potential therapeutic role and guide future research.

A comprehensive literature survey was conducted using the Scopus, PubMed, ScienceDirect, Web of Science, and Google Scholar databases using the term “*Ruscus aculeatus*” or “butcher’s broom”. Studies for which the full text was not available in English were excluded, as were studies in which the reported biological activity could not be attributed solely to *R. aculeatus* but rather to multi-component mixtures containing the plant.

## 2. Botanical Description

**Figure 1:**
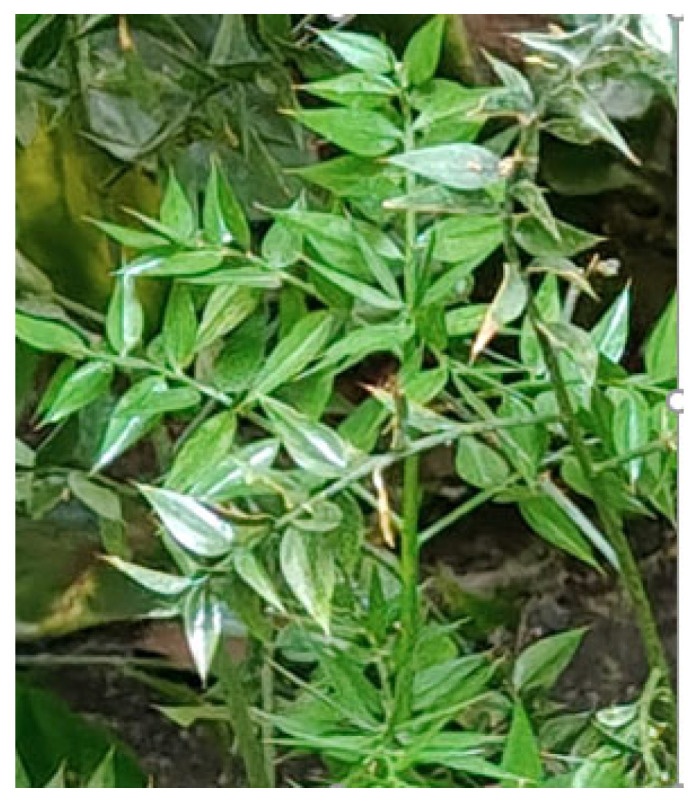
Photograph of the *Ruscus aculeatus* plant taken at the Botanical Garden, UMCS, Lublin, Poland.

*Ruscus aculeatus* (Figure 1) is an evergreen, perennial herbaceous species, growing up to 80 cm. The representatives of this species are sometimes described as subshrubs, or shrubs; however, the shoots of *Ruscus* are not woody. It develops a short, underground rhizome forming dense clusters, which represents the pharmacognostically relevant part of the plant (*Ruscus* rhizoma) [2]. The aerial shoots are unbranched and bear green, rigid phylloclades—flattened, leaf-like branches arising from the axils of reduced leaves. These phylloclades, ovate to lanceolate, leathery, and ending in a sharp spine, perform an assimilative function [5,21]. True leaves occur only in seedlings or occasionally directly from the rhizome. Small, sessile bracts subtend the solitary, dioecious flowers located on the lower surface of the phylloclades [2]. The flowers are small and yellowish-white, with six pale green tepals, and either a violet ovary or stamens (the species is dioecious). Flowers develop between March and April. Butcher’s broom produces bright red berries, ripening in the autumn and winter, up to 15 mm in diameter, each containing one or two seeds [5,22]. The combination of an underground rhizome and phylloclades with axillary flowers and spiny tips is characteristic and allows easy identification of *R. aculeatus* in pharmacognostic analysis.

## 3. Phytochemistry

### 3.1. Steroidal Saponins

**Figure 2:**
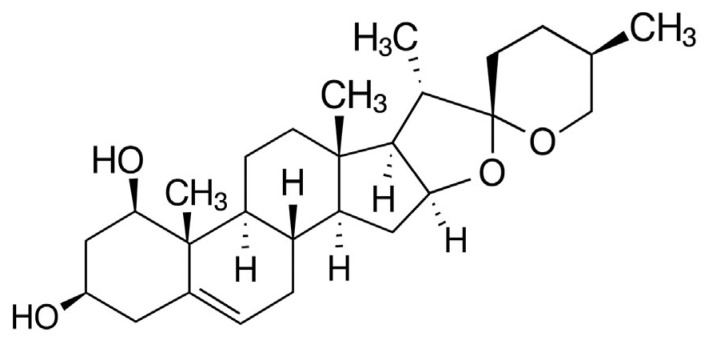
Chemical structure of ruscogenin.

Phytochemical investigations of *R. aculeatus* have revealed a complex chemical profile dominated by steroidal saponins of both the spirostanol and furostanol types. In general, steroidal saponins possess a C_27_ carbon skeleton, usually derived from oxidized cholesterol, and contain one or more sugar residues attached at various positions. Spirostanol saponins are characterized by a bicyclic ketal structure at C-22, whereas furostanol saponins typically contain a hemiketal group at C-22 and an additional sugar moiety (most often a single β-d-glucose unit) linked to C-26. The principal aglycone of the spirostanol saponins identified in *R. aculeatus* is ruscogenin (Figure 2).

Among the numerous glycosidic derivatives isolated from the underground parts are ruscoside, ruscin, deglucoruscin, deglucoruscoside, desglucoruscoside, and a series of complex saponins such as ruscoponticosides (C–E), ceparosides (A, B), aculeosides (A, B), and spilacleosides (A, B), often containing sugar chains composed of α-l-rhamnose, β-d-galactose, and β-d-glucose units, variably acetylated at positions 3, 4, or 6 [23,24,25,26]. A characteristic feature of several metabolites is the presence of sulfated moieties [27,28]. The saponin profile also includes glycosides with molecular formulas ranging from approximately C_39_H_62_O_13_ to C_58_H_92_O_27_, comprising mono-, di-, and tri-acetylated sugar chains and furostanol-type derivatives glycosylated at C-26 [29,30].

Because the structure of steroidal saponins is very complex, hydrolysis is often performed to release free aglycones. Alkaline hydrolysis with KOH under elevated temperature conditions (4 h) yielded stigmasterol, ruscogenin, and neoruscogenin [31]. Ruscogenin was also identified following acid hydrolysis with 1% sulfuric acid for 12 h [32]. Free ruscogenin and neoruscogenin were also detected after treatment with 37% HCl (heating for 1 h) or 1 M H_2_SO_4_ (heating for 8 h) [33,34].

Interestingly, tissue culture systems (in vitro callus and regenerant cultures) of *R. aculeatus* retain the biosynthetic capacity to produce the same phytochemical markers found in the natural plant, including ruscogenin, neoruscogenin, ruscoside, ruscin, and desglucoruscin. Their qualitative and quantitative composition can be modulated by culture conditions and elicitation with methyl jasmonate [35,36,37].

### 3.2. Phenolic Compounds

Beyond saponins, *R. aculeatus* contains a diverse array of phenolic compounds and flavonoids, particularly in its aerial parts. HPLC–MS and UHPLC–MS/MS analyses identified derivatives of apigenin, quercetin, and kaempferol, including apigenin-*C*-Hexoside-*C*-pentoside, quercetin-*O*-deoxyhexoside-hexoside, caffeic acid hexoside, and kaempferol-*O*-deoxyhexoside-hexoside, found in hydroalcoholic extracts prepared from stems and rhizomes [38,39,40]. In the berries, anthocyanins were detected, primarily pelargonidin derivatives such as pelargonidin-3-glucoside, pelargonidin-3-rutinoside, and pelargonidin-3-*p*-coumaroylglucoside [41].

### 3.3. Other Minor Components

Additionally, ethanol and chloroform extracts of rhizomes yielded non-saponin constituents, including 12-docosenoic acid and euparone [42,43]. More recent studies reported the presence of phenethylamine-type alkaloids, such as aculebiphenyl A and aculebiphenyl B, together with mesembrine and mesembrenone [44].

Overall, the phytochemical profile of *R. aculeatus* represents a rich spectrum of spirostanol and furostanol saponins—often sulfated and/or acetylated—complemented by polyphenols, anthocyanins, fatty acids, and minor alkaloids. However, quantitative data are limited. Some studies report content per dried extract or as isolation yields [26,28,38,39], which does not reflect total plant content. Only a few studies provide quantitative data, but these results should be interpreted with caution, as compound identification was based solely on retention time comparison with standards. It has been reported that the contents of *p*-coumaric acid and quercetin in the aerial parts of the plant are 3.0 and 4.1 mg/g, respectively [40]. The ruscogenin content in the aerial and underground parts of four *Ruscus* taxa ranged from 0.06 to 0.22% in the herb and 0.04 to 0.19% in the rhizome [45]. Walasek-Januszet al. reported a content of rusogenin in the range of 0.3–0.38 and of neorusogenin in the range of 0.83–1.02% in rhizomes [46]. In turn, Tansi et al. found that the sum of ruscogenin and neoruscogenin in the aerial parts was 0.03–0.05%, and in the underground parts, it was 0.02–0.12% [47].

The main findings regarding the phytochemical constituents of *R. aculeatus* are summarized in Table 1.

## 4. Biological Activity

### 4.1. Venotonic and Vasoprotective Effects

*R. aculeatus* is best known for its venotonic and vasoprotective effects. In this context, it is frequently mentioned in numerous review articles discussing the use of herbal drugs in chronic venous disease (CVD) [16,17,54,55,56].

CVD is a pathological condition caused by venous blood stasis resulting from incompetence of the venous valves. The disease is very common in adults and is responsible for skin changes and for approximately 40% of venous leg ulcers [57,58]. In the initiation of primary CVD, the process begins with leukocyte adhesion, followed by degranulation and migration beneath the venous endothelium, leading to chronic inflammation and subsequent remodeling of the venous valves and wall [59]. In the development of secondary CVD, persistent vein obstruction or recurrent deep vein thrombosis results in valve damage and venous hypertension [60]. This leads to increased leukocyte adhesion, excessive transcapillary filtration, and edema. Dilated, tortuous capillaries with impaired flow reduce oxygen delivery, leading to hyperpigmentation and lipodermatosclerosis [61]. In advanced CVD, fluid accumulation and venous abnormalities manifest as telangiectasias, varicose veins, pigmentation changes, pain, cramps, and restless legs [62].

Scientific reports verifying the venoactive and vasoprotective properties of *R. aculeatus* alone are scarce and originate mainly from the 1980s and 1990s. In turn, most recent studies have focused on combinations—particularly the mixture of *R. aculeatus*, hesperidin methyl chalcone (HMC), and vitamin C (Cyclo 3 Fort; Pierre Fabre, Paris, France). This formulation was developed in 1959 and is currently used as a first-line treatment for alleviating CVD symptoms. However, this review focuses specifically on the activity of *R.* aculeatus alone and will not discuss this combination, as several review articles have already addressed the topic in detail [20,56,63].

Regarding *R. aculeatus*, several studies have examined its impact on the vascular system. However, these studies are outdated, and many lack detailed information. A critical review of these papers was conducted in a report by the Committee on Herbal Medicinal Products (EMA/HMPC/188805/2017) [15]. The main directions of the investigated activities included venous and lymphatic contractility, vascular permeability, and effects on the endothelium.

Overall, the available studies consistently indicate that the extract of *R. aculeatus* exerts a contractile effect on venous vessels. In vitro studies demonstrated that its venoconstrictive effect is concentration-dependent and is mediated both by activation of postjunctional α_1_- and α_2_-adrenergic receptors and by stimulation of norepinephrine release. The response was shown to be independent of the endothelium and depended on temperature—it was enhanced by warming and inhibited by cooling [64,65,66,67,68]. The activity of *R. aculeatus* was further confirmed in in vivo models [69,70].

Furthermore, the study by Nemcova et al., based on veins collected from patients with primary varicose veins, demonstrated that extract of *R. aculeatus* exerts venotonic effects through modulation of cyclic nucleotide signaling in venous tissue. Experiments comparing varicose and non-varicose human saphenous veins showed that the extract selectively increased intracellular cAMP levels without affecting cGMP concentrations. This response was associated with normalization of the prostacyclin/thromboxane ratio, suggesting a regulatory influence on vascular tone and endothelial function [71].

The main feature of CVD is increased permeability of the venous endothelium, which leads to edema. Studies have demonstrated that *R. aculeatus* exerts a contractile effect on lymphatic vessels, thereby enhancing lymphatic flow [72]. Furthermore, it inhibits microvascular permeability. This effect was demonstrated in a hamster cheek pouch model, and the response was mediated by calcium and α_1_-adrenoreceptors [69]. These findings were further supported by in vivo experiments in anesthetized cats, where intravenous or oral administration of the extract prior to edema induction significantly reduced protein content and water accumulation in edematous tissue [73]. Moreover, a recent study showed that the extract can inhibit permeability changes induced by both histamine and ischemia/reperfusion. It also reduced leukocyte–endothelium interactions. In most assays, the *Ruscus* extract exhibited greater activity than diosmin [74]. The anti-edematous activity of *Ruscus* extract is most likely attributable to spirostanol saponins. Barbič et al. investigated the effects of isolated compounds from the rhizome on thrombin-induced hyperpermeability in human microvascular endothelial cells and found that spirostanol saponins and esculin reduced endothelial hyperpermeability. The strongest effects were observed for deglucoruscin, ruscin, and esculin at a concentration of 100 µM, with reductions of 41.9%, 42.6%, and 53.3%, respectively [50].

Another important mechanism of *R. aculeatus* involves its effects on the vascular endothelium. Experimental findings indicate that *R. aculeatus* can counteract hypoxia-induced endothelial activation. The extract inhibited the hypoxia-induced decrease in ATP content, the activation of phospholipase A_2_, and increase in neutrophil adhesion. Additionally, *Ruscus* extract and HMC exhibited additive effects in preventing ATP depletion [75]. In turn, Lestienne et al. reported that *Ruscus* extract exhibits binding affinity for muscarinic receptors, acting as a partial agonist, particularly at the M1 and M3 subtypes. This muscarinic activity appears to contribute to its vasoprotective and anti-inflammatory effects by reducing vascular permeability and leukocyte–endothelium interactions [76].

It should be mentioned that in most reports, the *Ruscus* extracts were not standardized, their chemical composition was not provided, and no information was given on how the extracts were prepared; therefore, the interpretation and comparison of the results should be made with caution. Table 2 summarizes the main studies on the effects of *R. aculeatus* on the venous and lymphatic system.

It is worth emphasizing that only one clinical study has evaluated the efficacy of *Ruscus* extract alone in CVD. The study, which included 166 women, showed that *R. aculeatus* extract significantly reduced leg volume, as well as ankle and lower leg circumference, during the study. In addition, the extract alleviated subjective symptoms such as heaviness, tiredness, tension, and tingling in the legs [77].

Beyond its phlebotherapeutic effects, *R. aculeatus* also appears to relieve symptoms of orthostatic hypotension [78]. Furthermore, it may be beneficial in diabetic retinopathy, a condition in which the small blood vessels of the retina are damaged due to elevated glucose levels. Evaluation of the fundus of the eye in patients treated with *Ruscus* showed a clear improvement in 23.1% of cases and slight improvement in visual field sensitivity after three months of therapy. Treatment significantly reduced blood glucose, fructosamine, glycosylated hemoglobin, and total cholesterol while raising HDL cholesterol. These findings suggest that *Ruscus* extract exerts a beneficial influence on both metabolic and vascular parameters in diabetic patients [79].

### 4.2. Antimicrobial Effects

Research on the antimicrobial activity of *R. aculeatus* is scarce, with only four studies addressing this topic to date. These works, summarized in Table 3, focus primarily on alcoholic extracts or infusions prepared from both aerial and underground parts of the plant. Unfortunately, most reports do not provide the phytochemical composition of the extracts, and in the case of the aerial parts, they do not clearly specify which plant sections were used. The only exception is the study by Rodrigues et al. [38], who characterized the phenolic composition of extracts from the aerial parts and specified that cladodes or laminar stems and lateral branches were used for extract preparation. They tested hydroethanolic extracts (80:20, *v*/*v*), infusions, and decoctions from aerial parts and underground organs against eight bacterial strains [38]. Preparations from the roots showed similar activity, with minimal inhibitory concentrations (MICs) of 20 mg/mL or higher. Only the decoction exhibited greater efficacy against MRSA (MIC = 10 mg/mL) compared to the ethanolic extract and infusion. These root preparations were also less effective than those obtained from aerial parts, for which MIC values ranged from 5 to >20 mg/mL.

Hadžifejzović et al. examined a 70% methanolic extract of rhizomes and aerial parts, as well as the fractions obtained by partitioning the herb extract. In general, all extracts exhibited similar antimicrobial activity, with no significant differences. The ethyl acetate fraction demonstrated stronger antibacterial activity against the five tested bacterial strains compared to the butanol fraction and exhibited superior antifungal activity against all tested fungi, except *Aspergillus niger* [80]. However, the weakness of the paper lies in the characterization of the phenolic composition of the extract. The authors refer to unpublished research results that were described in a doctoral dissertation (not accessible).

Ali-Shtayeh et al. investigated the antifungal activity of a water extract from the aerial parts of the plant. At 15 µg/mL, the extract showed significant antimycotic effects against several dermatophyte species, including *Microsporum canis*, *Trichophyton mentagrophytes*, and *Trichophyton violaceum*, with mycelial inhibition percentages of 83.6% (MIC = 35 µg/mL), 86.3% (MIC = 29 µg/mL), and 100% (MIC = 15 µg/mL), respectively [81]. In another study, the same authors evaluated the antimicrobial activity of an ethanolic extract and an infusion. The infusion demonstrated more pronounced activity than the ethanolic extract, which inhibited the growth of only two out of the six tested strains. In contrast, the infusion inhibited five bacterial species, with the exception of *Klebsiella pneumoniae* [8]. In turn, Festa et al. investigated the antimicrobial activity of saponins isolated from the underground parts of the plant. Among the 15 isolated components, only deglucoruscin showed mild activity. At a concentration of 128 µg/mL, it reduced the growth of *S. aureus* by approximately 30–36%, and it inhibited the growth of *C. albicans* by 31% at 64 µg/mL. Interestingly, the compound showed strong synergistic effects when combined with conventional drugs [52].

Furthermore, Sanna et al. evaluated the antiviral potential of *R. aculeatus*. The ethyl acetate fraction, obtained from the acetone extract of *R. aculeatus* leaves through sequential solvent extraction (n-hexane, ethyl acetate, acetone, and methanol), was tested against several RNA and DNA viruses, including Coxsackievirus B5 (CVB-5), Human Immunodeficiency Virus type 1 (HIV-1), Bovine Viral Diarrhea Virus (BVDV), and Yellow Fever Virus (YFV). The extract demonstrated notable activity against BVDV (EC_50_ = 58 µg/mL) and YFV (EC_50_ = 66 µg/mL), without cytotoxic effects on the Madin–Darby Bovine Kidney (MDBK) and Baby Hamster Kidney (BHK) host cell lines [82].

In addition, a recent study has shown that ethanolic extract from the rhizome possesses immunomodulatory and antimicrobial properties in skin cells. In primary human keratinocytes, the extract was found to induce the expression of the antimicrobial peptide RNase 7 through activation of the ERK signaling pathway, without affecting cell viability or triggering an inflammatory response. This suggests that *R. aculeatus* may enhance innate skin defense mechanisms under noninflammatory conditions [83].

**Table 3:** Antibacterial and antifungal activity of extracts from *R. aculeatus*.

Part of Plant/Extract	Antibacterial/Antifungal Effect	Ref.
roots and rhizomes/ maceration with stirring 1 h using 80% ethanol (e), infusions (i), or decoctions (d)	MIC/MBC (mg/mL) *Escherichia coli*: 20/>20 (e, d), >20/>20 (i) *Klebsiella pneumoniae*: >20/>20 (e, i, d); *Morganella morganii*: >20/>20 (e, i, d); *Proteus mirabilis*: >20/>20 (e, i, d); *Pseudomonas aeruginosa*: >20/>20 (e, i, d); *Enterococcus faecalis*: 20/>20 (e, i), >20/>20 (d); *Listeria monocytogenes*: >20/>20 (e, i, d); MRSA: >20/>20 (e), 20/>20 (i), 10/>20 (d)	[38]
rhizome/ 70% MeOH	MIC/MBC or * MIC/MFC (mg/mL) *Staphylococcus aureus*: 0.2/0.5; *Bacillus cereus*: 0.5/1.0; *Micrococcus flavus*: 1.0/2.0; *Listeria monocytogenes*: 1.0/1.0; *Pseudomonas aeruginosa*: 0.2/0.5; *Enterobacter cloacae*: 1.0/1.0; *Salmonella typhimurium*: 0.2/0.5; *Escherichia coli*: 0.5/1.0; *Trichoderma viride* *: 1.0/2.0; *Penicillium funiculosum* *: 0.5/2.0; *Aspergillus fumigatus* *: 2.0/3.0; *Aspergillus niger* *: 2.0/2.0; *Aspergillus versicolor* *: 2.0/3.0	[80]
aerial parts/infusion for 72 h	*Microsporum canis*: MIC 35 µg/mL *Trichophyton mentagrophytes* MIC 29 µg/mL, *Trichophyton violaceum* MIC 15 µg/mL	[81]
aerial parts/infusion (w) maceration with 95% ethanol (e) tested concentration: 200 mg/mL	ZOI (mm); disk diameter = 6 mm *Staphylococcus aureus*: 9.9 (w), 7.5 (e); *Escherichia coli*: 8.0 (w), 6.0 (e); *Klebsiella pneumoniae*: 6.0 (w), 6.0 (e); *Proteus vulgaris*: 11.0 (w), 9.0 (e); *Pseudomonas aeruginosa*: 8.5 (w), 6.0 (e); *Candida albicans*: 7.6 (w), 6.0 (e)	[8]
aerial part/maceration with stirring 1 h using 80% ethanol (e) infusions (i) decoctions (d)	MIC/MBC mg/mL *Escherichia coli*: 10/>20 (e), >20/>20 (i), 20/>20 (d); *Klebsiella pneumoniae*: 20/>20 (e, i, d); *Morganella morganii*: 10/>20 (e, i), 20/>20 (d); *Proteus mirabilis*: 20/>20 (e), >20/>20 (i, d); *Pseudomonas aeruginosa*: >20/>20 (e, i, d); *Enterococcus faecalis*: 10/>20 (e, i), 20/>20 (d); *Listeria monocytogenes*: 10/>20 (e, i, d); MRSA: 10/>20 (e, i), 5/>20 (d)	[38]
herb/70% MeOH (m)ethyl acetate fraction (ea)butanol fraction (b)	MIC/MBC or * MIC/MFC (mg/mL) *Staphylococcus aureus*: 0.1/0.2 (m, ea), 0.5/4.0 (b); *Bacillus cereus*: 1.0/4.0 (m, b), 0.5/2.0 (ea) *Micrococcus flavus*: 2.0/4.0 (m), 0.5/2.0 (ea), 2.0/2.0 (b); *Listeria monocytogenes*: 0.2/1.0 (m), 0.2/0.5 (ea), 1.0/2.0 (b); *Pseudomonas aeruginosa*: 1/4.0 (m), 0.5/2.0 (ea), 1.0/2.0 (b); *Enterobacter cloacae*: 1/4.0 (m), 0.5/2.0 (ea), 1.0/2.0 (b); *Salmonella typhimurium*: 0.2/0.5 (m, ea, b); *Escherichia coli*: 1.0/1.0 (m), 0.5/1.0 (ea, b); *Trichoderma viride* *: 1.0/2.0 (m), 0.25/0.5 (ea), 0.5/2.0 (b); *Penicillium funiculosum* *: 1.0/2.0 (m), 0.5/2.0 (ea, b); *Aspergillus fumigatus* *: 1.0/2.0 (m), 1.0/3.0 (ea, b); *Aspergillus niger* *: 2.0/2.0 (m), 1.0/3.0 (ea), 0.2/0.5 (b); *Aspergillus versicolor* *: 1.0/2.0 (m, ea), 1.0/3.0 (b)	[80]
aerial part/maceration with MeOH (40 °C, 3 h)tested concentration: 100 mg/mL	*Escherichia coli*: ZOI 6 mm *Klebsiella pneumoniae*: ZOI 10 mm *Staphylococcus aureus*: ZOI 15 mm *Candida albicans*: ZOI 15 mm	[84]

MRSA—methicyllin-resistant *Staphylococcus aureus*; MIC—minimum inhibitory concentration; ZOI—zone of inhibition (mm); MBC—minimum bactericidal concentration; MFC—minimum fungicidal concentration; *— for the fungal strains, MFC values were provided instead of MBC.

### 4.3. Antioxidant Effect

Antioxidant activity is one of the most extensively investigated biological effects of plant extracts, as antioxidants exert beneficial effects on human health both internally and externally. When consumed, they can neutralize free radicals, reduce oxidative stress, and support the proper functioning of the cardiovascular system, immune response, and metabolic processes. When applied topically, they may accelerate wound healing, protect the skin from oxidative damage, and delay premature aging by limiting the harmful effects of reactive oxygen species.

Overall, the available data indicate that *R. aculeatus,* including both aerial and underground parts, shows moderate to weak free radical scavenging activity (Table 4).

Jakovljević et al. investigated the antioxidant activity of three extracts (ethanolic, acetone, and ethyl acetate) obtained from the aerial parts of the plants and found that the results of the DPPH and ABTS assays were notably weaker compared to ascorbic acid (AA). Interestingly, all extracts exhibited significant reducing power and metal-chelating effects, with FRAP assay results even surpassing those of ascorbic acid (AA) [85]. Similarly, various extracts from the aerial parts of *R. aculeatus*, obtained through successive maceration and Soxhlet extraction with petroleum ether, chloroform, and ethanol, were evaluated by Taşkin et al. using DPPH, ABTS, FRAP, and CUPRAC assays. Soxhlet extraction yielded higher antioxidant activity than maceration; however, in agreement with other reports, the results were considerably weaker than those of the standards used as positive controls [86]. In turn, Rodrigues et al. [38] evaluated the antioxidant activity of hydromethanolic and aqueous extracts from both the aerial and underground parts of *R. aculeatus* using two complementary in vitro assays: (i) the Thiobarbituric Acid Reactive Substances (TBARS) formation inhibition capacity, reflecting the ability to prevent lipid peroxidation, and (ii) the OxHLIA assay, which measures the protective effect of extracts on erythrocytes against oxidative hemolysis. The hydroethanolic extract from the aerial parts exhibited the highest activity in the TBARS assay. In the OxHLIA assay, the most active extracts were the ethanolic root extract and the infusion from aerial parts, with comparable IC_50_ values of 230 µg/mL and 236 µg/mL (∆t = 60 min), respectively. Nevertheless, in both assays, *Ruscus* extracts demonstrated significantly lower activity than ascorbic acid and Trolox, which were used as positive controls.

**Table 4:** Antioxidant activity of extracts from *Ruscus aculeatus*.

Plant Part/Extract	Test (Control)	Ref.
Underground part/ 80% ethanol (e) Infusions (i) Decoctions (d)	TBARS EC_50_ (mg/mL): 0.78 (e), 1.0 (i), 1.55 (d) (Trolox: 0.0058) OxHLIA IC_50_ (µg/mL): ∆ t = 60 min 230 (e), 646 (i), 661 (d) (Trolox: 21.8) ∆ t = 120 min 383 (e), 1389 (i), 1198 (d) (Trolox: 43.5)	[38]
Rhizome/70% MeOH	DPPH IC_50_ (µg/mL): 386 (no control)	[80]
Aerial part/ Ethanolic (e) Ethyl acetate (ea) Acetone (a)	DPPH IC_50_ (µg/mL): 502 (e), 182.5 (ea), 227.2 (a) (AA: 6.05) ABTS IC_50_ (µg/mL): 3.5 (e), 3.4 (ea), 3.4 (a) (AA: 2.85) FRAP IC_50_ (µg/mL): 209 (e), 223 (ea), 239 (a) (AA: 881) FIC IC_50_ (µg/mL): 150 (e), 165 (ea), 170 (a) (AA: 352.9) LPI (mg/mL): 1.0–0.79 (e), 1.05–0.81 (ea), 1.2–0.84 (a) (AA: 0.4–0.25)	[85]
Aerial part/ 80% ethanol (e) Infusions (i) Decoctions (d)	TBARS EC_50_ (mg/mL): 0.28 (e), 0.49 (i), 0.88 (d) (Trolox: 0.0058) OxHLIA IC_50_ (µg/mL): ∆ t = 60 min 0 (e), 236 (i), 427 (d) (Trolox: 21.8) ∆ t = 120 min 0 (e, i, d) (Trolox: 43.5)	[38]
Stems, leaves/methanol	DPPH IC_50_ (µg/mL): 171.9 (AA: 0.3)	[40]
Herb/70% MeOH (m) Ethyl acetate fraction (ea) Butanol fraction (b)	DPPH IC_50_ (µg/mL): 206 (m), 158 (ea), 173 (b) (no control)	[80]
Aerial part/successive Soxhlet: petroleum ether (pe), chloroform (chl), Ethanol (e)	DPPH IC_50_ (mg/mL): 2.32 (pe), 0.18 (chl), 0.26 (e) (AA: 0.005) ABTS (mM trox/g): 1.63 (pe), 1.92 (chl), 3.24 (e) (AA: 13.01) FRAP (mM Fe^2+^/mg): 0.15 (pe), 0.37 (chl), 0.12 (e), (BHT: 1.1) CUPRAC (mM trolox/mg): 0.35 (pe), 0.86 (chl), 0.1 (e), (BHA: 1.62)	[86]
Aerial part/successive maceration: petroleum ether (pe), chloroform (chl), ethanol (e)	DPPH IC_50_ (mg/mL): 0.83 (pe), 0.81 (chl), 0.79 (e) (AA: 0.005) ABTS (mM trox/g): 1.56 (pe), 3.1 (chl), 3.22 (e) (AA: 13.01) FRAP (mM Fe^2+^/mg): 0.1 (pe), 0.33 (chl), 0.86 (e), (BHT: 1.1) CUPRAC (mM trox/mg): 0.25 (pe), 0.5 (chl), 0.15 (e), (BHA: 1.62)	[86]
Shoot/decoctions (d), maceration: 40% ethanol (e40), 96% ethanol (e96)	ABTS (mmol trox/100 g DW): 0.3 (d), 1.7 (e40), 0.1 (e96) DPPH (mmol trox/100 g DW): 0.1 (d), 2.2 (e40), 0.1 (e96) FRAP (mmol FeSO_4_^+^ DW): 1.2 (d), 4.8 (e40), 0.8 (e96)	[39]
Aerial parts/maceration with methanol (40 °C, 3 h)	TPC (mg GAE/g): 229.7 DPPH IC_50_ (mg/mL): 0.209 (BHA: 0.009, BHT: 0.365) β-CAR–LA (% inhibition): 58.6 (BHA: 66.08, BHT: 67.9)	[84]

FIC—ferrous ion chelating; LPI—inhibitory activity against lipid peroxidation during 48–96 h; AA—ascorbic acid; BHT—butylated hydroxytoluene; BHA—butylated hydroxyanisole; trox—trolox; GAE—gallic acid equivalent; β-CAR–LA—β-carotene–linoleic acid assay.

### 4.4. Anticancer Activity

Research on the anticancer properties of *R. aculeatus* remains limited, and a major limitation of these studies is the lack of, or only partial, phytochemical characterization of the extracts. However, existing reports suggest that both aerial and underground parts of the plant possess cytotoxic potential (Table 5). Rodrigues et al. investigated the anticancer activity of hydroethanolic extracts, decoctions, and infusions from the aerial and underground parts of the plant against several cancer cell lines, including HeLa (cervical carcinoma), NCI-H460 (non-small cell lung cancer), MCF-7 (breast adenocarcinoma), and HepG2 (hepatocellular carcinoma), using the sulforhodamine B (SRB) assay. They observed that most of the extracts effectively inhibited the growth of the tested cell lines, with the hydroethanolic extract of the aerial parts being the most effective. However, both the hydroethanolic extracts and the decoction of the underground parts of the plant also showed toxicity toward the primary liver cell culture (PLP2) [38].

In turn, Bassil et al. conducted extensive studies on the effects of ethanol extracts from the aerial parts of *R. aculeatus* on human acute T-cell lymphoblastic leukemia cells (Jurkat cell line). The cytotoxicity of each extract was assessed using lactate dehydrogenase (LDH) and MTT assays. *R. aculeatus* showed a cytotoxic effect on Jurkat cells, with IC_50_ values between 10 and 16 mg/mL at 24 h and between 8 and 10 mg/mL at 48 h (LDH). Unfortunately, *R. aculeatus* also exhibited a similar cytotoxic effect on normal lymphocytes. Furthermore, molecular studies evaluating apoptosis- and cell cycle-related pathways were performed. It was found that proteins directly involved in apoptosis (Caspase-8, Smac/DIABLO) and those indirectly involved (Bcl-2, Bax) did not show significant changes. *R. aculeatus* also did not significantly alter CDK-4 expression; however, it caused a decrease in p53 levels. Taken together, these results showed that *R. aculeatus* extract induces a non-apoptotic form of Jurkat cell death [87].

Bilušić et al. investigated the effect of a shoot water extract, prepared by heating at 100 °C for 5 min, on human bladder cancer (T24) and lung cancer (A549) cell lines, as well as a control line: the human embryonic kidney cell line HEK 293. The effects were evaluated at 4, 24, 48, and 72 h using the MTT assay. For T24 and A549 cells, a time-dependent increase in cancer cell inhibition was observed. After 72 h, 35–38% inhibition was recorded at the highest tested concentration, compared to cisplatin (positive control), which caused approximately 50% inhibition in T24 cells and 13–14% in A549 cells. In turn, for HEK 293 cells, the highest cytotoxicity was only observed at 4 and 24 h, with 32–36% inhibition, while cisplatin showed no inhibition [88].

The effect of saponins isolated from the underground part on human promyelocytic leukemia HL-60 cells was evaluated using the MTT assay. Among the 12 tested saponins, a furostanol saponin with a diglycoside moiety modified by a (2*S*,3*S*)-2-Hydroxy-3-methylpentanoic acid group and an acetic acid group, as well as its corresponding spirostanol saponin, exhibited significant cytotoxic activity after 72 h of treatment, showing 92.4% and 98.2% inhibition at 10 μg/mL, respectively. The IC_50_ values of these two compounds were approximately 3.5 and 3.0 μg/mL, respectively. Mimaki et al. suggested that the acetyl and 2-Hydroxy-3-methylpentanoyl groups attached to the diglycoside moiety are crucial for the observed cytostatic activity [23,24].

### 4.5. Anti-Inflammatory Activity

Rodrigues et al. evaluated the anti-inflammatory activity of hydroethanolic extracts, decoctions, and infusions prepared from the aerial and underground parts of *R. asuleatus* by measuring NO levels in LPS-activated murine macrophages. Nitric oxide (NO) is widely used as a marker of inflammation, as it is synthesized by inducible nitric oxide synthase (iNOS) during immune responses. Among the tested extracts, only the hydroethanolic extract from the aerial parts and the decoction from the root and rhizome showed anti-inflammatory activity, with EC_50_ values of 60 and 129 μg/mL, respectively (compared to dexamethasone with an EC_50_ of 16 μg/mL) [38].

Furthermore, fractions of steroidal saponins isolated from the rhizomes were investigated for their anti-inflammatory activity using the rat paw edema model. The saponins were administered orally 1 h before induction of inflammation with carrageenan or kaolin. At a dose of 500 mg/kg, the saponins significantly reduced carrageenan-induced edema by 43–69% within 1–5 h, and the effect was comparable to or even greater than that of diclofenac. In the kaolin model, edema was reduced by 64.4% and 40% at 4 h and 24 h, respectively (compared to 52.1% and 41% for diclofenac) [89]. An in vivo study using rats with inflammation induced by turpentine oil also confirmed the anti-inflammatory effect of the saponin mixture on the acute-phase bone marrow response, phagocytic capacity, and total antioxidant response. It was found that oral administration of the saponins decreased the leukocyte count and the percentage of neutrophils, indicating a reduction in inflammation. Additionally, the treatment reduced the phagocytic index and increased the total antioxidant response (TAR) [90].

### 4.6. Other Biological Activities

There are also a few individual studies investigating other pharmacological activities of butcher’s broom.

#### 4.6.1. Anti-Urease and Anticholinesterase Effects

Taşkin et al. investigated the anti-urease and anticholinesterase activities of extracts from the aerial parts of *R. aculeatus*, obtained through successive maceration and Soxhlet extraction with petroleum ether, chloroform, and ethanol. They reported that both chloroform and ethanol extracts exhibited inhibitory effects on these enzymes. The ethanol extract showed the highest anti-urease activity, achieving approximately 29% urease inhibition at a concentration of 12.5 µg/mL (positive control: thiourea, 78.8%). Conversely, the chloroform extract demonstrated the strongest anticholinesterase activity, with 94.4% inhibition at 500 µg/mL, a value comparable to that of the reference inhibitor galantamine (96.5%) [86]. Unfortunately, they did not provide any information on the components of the extracts.

The anti-urease assay is particularly relevant in the context of *Helicobacter pylori* infection, as this bacterium is responsible for various gastrointestinal disorders. Urease is a key virulence factor of *H. pylori* because it hydrolyzes urea into ammonia, which neutralizes gastric acid and enables the pathogen to survive in the highly acidic environment of the stomach. Inhibiting urease activity can therefore reduce bacterial colonization and help prevent gastritis, peptic ulcers, and even gastric cancer associated with chronic *H. pylori* infection.

Similarly, the anticholinesterase activity assay is commonly used in the search for agents that protect against neurodegenerative disorders, particularly Alzheimer’s disease. Acetylcholinesterase catalyzes the breakdown of acetylcholine, a neurotransmitter essential for memory and learning. Inhibitors of this enzyme can elevate acetylcholine levels in the synaptic cleft, thereby enhancing cholinergic transmission, improving cognitive function, and potentially slowing disease progression.

#### 4.6.2. Diuretic Activity

The steroidal saponin fractions extracted from the rhizomes of *R. aculeatus* have been shown to exert a diuretic effect. After oral administration in rats, they increased urinary volume as well as the excretion of Na^+^, K^+^, and Cl^−^ ions. The resulting activity produced a less intense but more sustained diuretic response compared to furosemide, the reference diuretic drug. The highest activity was observed 24 h after administration. According to the authors, the mechanism of action may be at least partially tubular, involving a reduction in the reabsorption of water and electrolytes within the renal tubules [90].

#### 4.6.3. Anti-Osteoporotic Activity

Chakuleska et al. [91] reported that *R. aculeatus* steroidal saponins may mimic the activity of sex hormones and help reduce risk of osteoporosis. In their study, an extract was prepared by combining 80% ethanolic and aqueous fractions from the roots and rhizomes, standardized to contain 20% saponins. This extract was administered orally to estrogen-deficient female rats subjected to ovariectomy (OVX), and markers of bone turnover (osteocalcin, alkaline and acid phosphatase, β-CrossLaps) and calcium–phosphate homeostasis (Ca, P, 25-Hydroxy vitamin D, parathyroid hormone (PTH), and estradiol) were evaluated. After 10 weeks of treatment with *R. aculeatus* extract, the altered parameters were restored to control levels, showing effects comparable to diosgenin and alendronate, which served as positive controls. It is worth noting that, after this period, there were also no statistically significant differences between untreated OVX rats and the sham-operated group. However, statistically significant differences between untreated OVX and OVX rats treated with the extract were observed after 35 days of treatment, confirming its impact on these parameters. Moreover, radiographs of animals treated with higher doses of the extract showed no signs of bone mineral content (BMC) deformities or expansion, in contrast to the untreated OVX group. Histological analysis revealed that OVX rats treated with 100 and 200 mg/kg of the extract showed signs of regeneration of the disturbed bone structure. Furthermore, the authors found that malondialdehyde (MDA), an indicator of lipid peroxidation, was significantly elevated in OVX rats, whereas reduced glutathione (GSH), a key antioxidant, was decreased. Treatment with the extract significantly lowered MDA levels and restored GSH to near-control values in the liver, intestines, and bones, demonstrating the extract’s ability to prevent oxidative stress. Cell line assays on the human osteoblast-like SaOS-2 cells confirmed the lack of cytotoxicity of *R. aculeatus*. Moreover, at a concentration of 200 μg/mL, it even demonstrated a stimulatory effect on cell proliferation.

Molecular docking studies with estrogen receptors revealed that ruscogenin and neoruscogenin, the key constituents of *R. aculeatus*, formed stable complexes with both estrogen receptor alpha and beta. Their binding scores were comparable to those of estradiol and diosgenin, suggesting that ERA could be developed as a potential candidate for the prevention of postmenopausal osteoporotic complications.

### 4.7. Side Effects

There are only a few studies on the adverse effects of *Ruscus* extract, and they mostly involve isolated case reports. Sadarmin et al. described the case of a 39-year-old woman who developed diabetic ketoacidosis (DKA) five days after initiating therapy with *R. aculeatus.* The patient experienced four episodes of vomiting and one episode of diarrhea within a 48 h period. The authors suggested that the concurrent use of butcher’s broom and metformin was the most probable trigger of her metabolic deterioration and led to hospitalization [92]. Ramírez-Hernández et al. reported an allergic reaction after the application of topical preparations in a 34-year-old woman, which, as further tests showed, was associated with ruscogenin [93].

## 5. Biological Activity of Ruscogenin

It is widely believed that one of the main active ingredients of butcher’s broom responsible for its biological effects is ruscogenin (molecular formula: C_27_H_42_O_4_; IUPAC name: (1β,3β,25*R*)-spirost-5-ene-1,3-diol). Ruscogenin is a bioactive compound that has been isolated from the rhizome of *R. aculeatus*, the radix of *Ophiopogon japonicus*, and the bulb stems of *Allium cepa*. Ruscogenin is a steroidal saponin of the spirostan type [36,94]. Its structure consists of two oxygen-containing heterocycles connected through a spiroacetal carbon atom located on the D ring of the steroid core, and the hydroxyl group at C-10 is glycosylated with a sugar chain.

However, as demonstrated by a review of the literature on the phytochemistry of *R. aculeatus*, ruscogenin is present in this plant material as an aglycone only in minor amounts, whereas its various derivatives constitute the principal constituents. Nevertheless, this low content does not diminish its pharmacological relevance, as ruscogenin may be generated through metabolic transformation following oral administration of *Ruscus* extract. Therefore, its pharmacological activity will be briefly discussed below.

Ruscogenin exhibits a broad spectrum of biological activities, including antioxidant, anti-inflammatory, neuroprotective, and anticancer properties. It has been found that it can inhibit neutrophil activation [95] and modulate key inflammatory mediators. For example, in mice with gastric ulcers, ruscogenin significantly decreased the levels of TNF-α, IL-6, IL-8, lipid peroxidation (LPO), and myeloperoxidase (MPO) while enhancing the activities of glutathione (GSH) and glutathione peroxidase (GSH-Px) [96]. A decrease in inflammatory mediators, including the expression of NF-κB target genes such as ICAM-1, inducible nitric oxide synthase (iNOS), cyclooxygenase-2 (COX-2), tumor necrosis factor-α (TNF-α), and interleukin-1β (IL-1β), was also observed in the mouse model of experimental stroke [97]. Ruscogenin also mitigates IL-1β-induced cartilage destruction and inflammation in osteoarthritis [98,99] and LPS-induced lung injury by inhibiting inflammatory mediators [100]. Furthermore, it displays neuroprotective properties across several neurological disease models. In models of Parkinson’s disease, ruscogenin reduces oxidative stress and nitric oxide production, suppresses IL-1β and IL-6 expression, and promotes dopaminergic neuron survival. In vivo, ruscogenin lowers the levels of TNF-α, IL-1β, IL-6, COX-2, and iNOS while improving motor coordination and grip strength in mice [101]. It inhibits amyloid-β oligomerization and aggregation, a critical process in the pathogenesis of Alzheimer’s disease [102].

In models of acute lung injury, ruscogenin was found to reduce endothelial cell apoptosis [103] and stabilize pulmonary endothelial barrier integrity [104]. Interestingly, it has shown potential in mitigating SARS-CoV-2 E protein-induced cell death, suggesting possible therapeutic applications in the management of COVID-19 complications [105].

In a rat model of monocrotaline-induced pulmonary arterial hypertension, ruscogenin effectively attenuated vascular remodeling and inflammation, demonstrating significant endothelial-protective activity [106]. Moreover, it has been shown to improve cardiac function, reduce myocardial fibrosis, and alleviate morphological damage, further supporting its cardioprotective potential [107].

**Figure 3:**
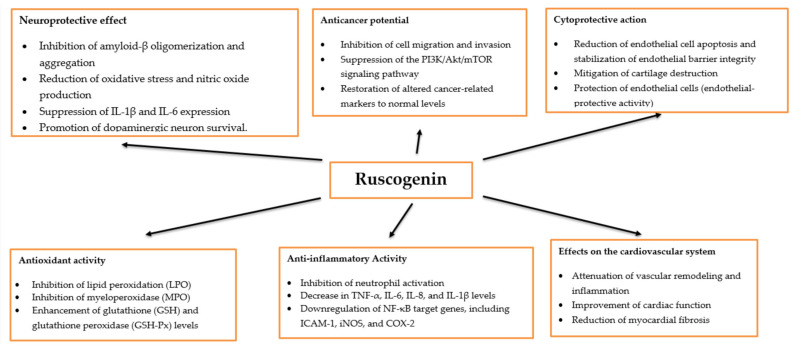
Examples of biological activities and related effects of ruscogenin.

Furthermore, ruscogenin demonstrates notable anticancer potential. Hua et al. reported that it inhibited the metastasis of hepatocellular carcinoma [108]. In another study using a lung cancer model, ruscogenin restored antioxidant balance, modulated immune responses, and normalized tumor-promoting markers to baseline levels [109]. The main directions of action of ruscogenin are presented in Figure 3.

## 6. Regulatory Status of *R. aculeatus*

Following the discussion of *R. aculeatus,* it is important to consider the regulatory frameworks that govern its use and marketing. Due to its broad range of potential applications, *R. aculeatus* is subject to various legal acts. Within the European Union (EU), the regulation of this plant depends on the product category. When incorporated into medicinal products, its use is governed by Directive 2001/83/EC on the Community code relating to medicinal products for human use [110] and by the Pharmaceutical Law [111]. In the case of cosmetic products, the relevant framework is Regulation (EC) No 1223/2009 of the European Parliament and of the Council of 30 November 2009 on cosmetic products [112]. When used in herbal medicinal preparations, *R. aculeatus* falls under Decision 2008/911/EC [113]. For its inclusion in dietary supplements, the applicable legislation includes Directive 2002/46/EC of the European Parliament and of the Council of 10 June 2002 on the approximation of the laws of the Member States relating to food supplements [114], together with Regulation (EC) No 1924/2006 on nutrition and health claims made on foods [115] and Regulation (EU) No 1169/2011 on the provision of food information to consumers [116]. *R. aculeatus* has been approved by the European Medicines Agency (EMA). The plant is also included in the European Pharmacopoeia and is subject to regulations governing herbal medicinal products, including registration as a Traditional Herbal Medicinal Product (THMP).

In the United States, the regulation of pharmaceuticals, cosmetics, and other related products falls under the jurisdiction of the Food and Drug Administration (FDA) [117]. Prior to marketing, products must receive approval depending on their classification and associated risk level. However, *Ruscus* has not yet been reviewed by the FDA.

Overall, *R. aculeatus* represents a species with applications spanning pharmaceuticals, dietary supplements, herbal medicines, and cosmetics. The selection of the appropriate legal framework depends on the intended application, and understanding these regulations clarifies how herbal medicines are standardized, labeled, and evaluated for safety and efficacy across jurisdictions.

## 7. Conclusions and Future Perspectives

*R. aculeatus* L. remains an important medicinal plant with a long history of traditional use. A review of the existing literature confirms its broad biological activities, including venoactive, vasoprotective, anti-inflammatory, antioxidant, and antibacterial effects, which justify its ethnomedicinal applications and indicate its relevance in modern phytotherapy. However, this review has also revealed several limitations, such as the scarcity of recent, well-designed studies investigating *R. aculeatus* as a single botanical entity. Moreover, toxicological and pharmacokinetic data for *Ruscus* extract and its major saponins remain incomplete, with limited information regarding its metabolism, bioavailability, and potential drug interactions. Many older studies also lack phytochemical standardization in terms of chemical composition, solvent system, extraction method, and quantification of active compounds. Furthermore, there has been limited exploration of molecular mechanisms underlying its biological effects.

Future research should therefore focus on the following: (I) comprehensive phytochemical profiling of standardized extracts using advanced analytical techniques (e.g., LC–MS/MS, NMR); (II) mechanistic studies aimed at elucidating the molecular targets and signaling pathways of steroidal saponins; (III) well-controlled preclinical experiments employing chemically defined preparations; and (IV) rigorous clinical trials assessing the efficacy, safety, and pharmacokinetics of *R. aculeatus* extracts or purified constituents.

In conclusion, *R. aculeatus* holds considerable pharmacological promise, but further research is warranted to fill the existing knowledge gaps. The use of modern phytochemical and pharmacological approaches will be essential to fully substantiate its therapeutic potential and ensure its safe, evidence-based application in contemporary medicine

## Figures and Tables

**Table 1:** Main chemical constituents found in *R. aculeatus* and identification techniques.

Extraction Solvent	Technique	Identified Components	Ref.
**Root/rhizome of *R. aculeatus***			
methanol	prep-HPLC, FABMS, C NMR	1/3-Hydroxyruscogenin 1-sulphate; 26-*O*-/3-d-Glc furost-5-en-1/3,22′-triol 1-sulphate	[27]
isolation by fractionation of methanolic extract	HPLC, NMR, FABMS	(23*S*,25*R*)-spirost-5-ene-3β,23-diol 23-*O*-{*O*-β-d-Glc-(1→6)-β-d-Glc}	[29]
methanol	HPLC–MS/MS	Deglucoruscoside; (25*R*)-furost-5-ene-1β,3β,22ξ,26-tetrol 1-*O*-[α-l-Rha-(1→2)-6-*O*-acetyl-β-d-Gal]-26-*O*-β-d-Glc; (25*R*)-spirost-5-ene-1β,3β-diol 1-*O*-[α-l-Rha-(1→2)-β-d-Gal]; ruscoponticoside C; (25*R*)-spirost-5-ene-1β,3β-diol 1-*O*-[α-l-Rha-(1→2)-6-*O*-acetyl-β-d-Gal]	[48]
isolation by fractionation of methanol extract	HPLC-MS, NMR, FABMS	Ruscogenin 1-*O*-{*O*-α-l-Rha-(1→2)-β-d-Gal}; Ruscogenin 1-*O*-{*O*-α-l-Rha-(1→2)-6-*O*-acetyl-β-d-Gal}; Ruscogenin 1-*O*-{*O*-α-l-Rha-(1→2)-4,6-di-*O*-acetyl-β-d-Gal}; Ruscogenin 1-*O*-{*O*-α-l-Rha-(1→2)-3,4,6-tri-*O*-acetyl-β-d-Gal}; Ruscogenin 1-*O*-{*O*-β-d-Glc-(1→3)-*O*-α-l-Rha-(1→2) -β-d-Gal}; 26-*O*-β-d-Glc-22-*O*-methyl-(25*R*)-furost-5-ene-1β,3β,22ξ,26-tetrol 1-*O*-{*O*-α-l-Rha-(1→2)-β-d-Gal}	[23]
30% ethanol	HRESI-MS, NMR	Spilacleosides A and B	[49]
isolation by fractionation of 70% ethanol extract.	HPLC–ESI/ITMS HPLC–UV HPLC–IR, NMR	Ruscoside; ruscoponticoside E; ruscoponticoside C; ruscoponticoside D ceparoside B; ceparoside A; 26-*O*-β-d-Glc-22α-methoxy-furosta-5,25(27)-diene-1β,3β,26-triol 1-*O*-[β-d-Glc-(1→3)-*O*-α-l-Rha-(1→2)-*O*-α-l-Ara]; 26-*O*-β-d-Glc-furosta-5,20(22),25(27)-triene-1β,3β,26-triol 1-*O*-[α-l-Rha-(1→2)-*O*-α-l-Ara]; 26-*O*-β-d-Glc-22α-methoxy-furosta-5,25(27)-diene-1β,3β,26-triol 1-*O*-[α-l-Rha-(1→2)-*O*-α-l-Ara]; 26-*O*-β-d-Glc-22α-methoxy-furosta-5,25(27)-diene-1β,3β,26-triol 1-*O*-[α-l-Rha-(1→2)-3-acetyl-4-[(2*S*,3*S*)-2-Hydroxy-3-methylPen]-α-l-Ara]; (25*R*)-Spirost-5-ene-1β,3β-diol 1-*O*-[α-l-Rha-(1→2)-*O*-α-l-Ara]	[25]
isolation by fractionation of 95% ethanol extract	HPLC, NMR, HR-ESI-MS,	Aculebiphenyl A, Aculebiphenyl B, mesembrine, mesembrenone	[44]
isolation by fractionation of methanol extract	sPrep-HPLC, TLC, NMR HR-ESI-MS,	Deglucoruscin, 22-*O*-methyl-deglucoruscoside, deglucoruscoside, ruscin, ruscogenin-1-*O*-[α-l-Rha-(1→2)-β-d-Gal], 1-*O*-sulpho-ruscogenin, 30-*O*-acetyl-4′-*O*-sulphodeglucoruscin, 4′-*O*-(2-Hydroxy-3-methylpentanoyl)-deglucoruscin, 4′-*O*-acetyl-deglucoruscin, esculin	[50]
isolation by fractionation of 60% ethanol extract	HPLC-UV; HPLC-ESI-MS, IR, NMR	Neoruscogenin; Ruscogenin; Ruscin; Desglucoruscin; Desglucodesrhamnoruscin; Ruscoside; Desglucoruscoside; 1-*O*-[α-l-Rha-(1→2)-6-*O*-acetyl-β-d-Gal]-1β,3β,22ξ,26-tetrahydroxy-furost-5(6)-en-26-*O*-β-d-Glc	[30]
isolation by fractionation of methanol extract	HPLC, MS, NMR	(23*S*)-spirosta-5,25(27)-diene-1β,3β,23-triol 1-*O*-{*O*-β-d-Glc-(1→3)-*O*-α-l-Rha-(1→2)-α-l-Ara} 23-*O*-β-d-Glc; (23*S*)-spirosta-5,25(27)-diene-1β,3β,23-triol 1-*O*-{*O*-α-l-Rha-(1→2)-α-l-Ara} 23-*O*-β-d-Glc	[51]
fractionation of methanol extract,	TLC, HPLC, NMR	Aculeoside A; Aculeoside B	[26]
isolation by fractionation of methanol extract	TLC, DCCC, HPLC, GC, HRESI-MS, NMR	26-*O*-β-d-Glc-furosta-5,25(27)-diene-1β,3β,22α,26-tetrol 1-*O*-[α-l-Rha-(1″→2′)-*O*-(3′,4′-di-*O*-acetyl)-α-l-Ara]; 26-*O*-β-d-Glc-22α-methoxy-furosta-5,25(27)-diene-1β,3β,26-triol 1-*O*-[α-l-Rha-(1″→2′)-*O*-(3′,4′-di-*O*-acetyl)-α-l-Ara]; 26-*O*-β-d-Glc-furosta-5,25(27)-diene-1β,3β,22α,26-tetrol 1-*O*-sulphate; (25*R*)-26-*O*-β-d-Glc-furost-5-ene-1β,3β,22α,26-tetrol 1-*O*-sulphate; Ceparoside A; Ruscoponticoside E; Ceparoside B; 26-*O*-β-d-Glc-furosta-5,20(22),25(27)-triene-1β,3β,26-triol 1-*O*-[α-l-Rha-(1→2)-*O*-α-l-Ara]; Spirosta-5,25(27)-diene-1β,3β-diol 1-*O*-[α-l-Rha-(1→2)-*O*-α-l-Ara]	[28]
isolation by fractionation of hydroalcoholic extract	DCCC, HPLC, NMR, MS	26-*O*-β-d-Glc-furosta-5,20(22),25(27)-triene-1β,3β,26-triol 1-*O*-α-l-Rha-(1→2)-4-[(2*S*,3*S*)-2-Hydroxy-3-methylPen]-α-l-Ara; 26-*O*-β-d-Glc-furosta-5,20(22),25(27)-triene-lβ,3β,26-triol 1-*O*-{*O*-α-l-Rha-(1→2)-α-l-Ara}; 26-*O*-β-d-Glc-22-*O*-methylfurosta-5,25(27)-diene-1β,3β,22ε,26-tetrol 1-*O*-{*O*-α-l-Rha-(1→2)-3-*O*-acetyl-4-*O*-[(2*S*,3*S*)-2-Hydroxy-3-methylPen]-α-l-Ara}; Ruscoponticoside C, E; Ruscoside; 26-*O*-β-d-Glc-22-*O*-methyl-furosta-5,25(27)-diene-1β,3β,22α,26-tetrol 1-*O*-[α-l-Rha-(1→2)-α-l-Ara]; 26-*O*-β-d-Glc-22-*O*-methyl-furosta-5,25(27)-diene-1β,3β,22α,26-tetrol 1-*O*-[α-l-Rha-(1→2)-4-*O*-[(2*S*,3*S*)-2-Hydroxy-3-methylPen]-α-l-Ara]; 2.3.11.26-*O*-β-d-Glc-22-*O*-methyl-furosta-5,25(27)-diene-1β,3β,22α,26-tetrol 1-*O*-[α-l-Rha-(1→2)-3-*O*-acetyl-4-*O*-[(2*S*,3*S*)-2-Hydroxy-3-methylPen]-α-l-Ara]; Nolinofuranoside G; 25*R*),26-*O*-β-d-Glc-22-*O*-methyl-furost-5-ene-1β,3β,22α,26-tetraol 1-*O*-sulphate]; Desglucodesrhamnoruscin; 4′-*O*-(2-Hydroxy-3-methylPen)-deglucoruscin; Ruscin; [(25*R*)-3β-hydroxyspitost-5-en-1β-yl *O*-α-l-Rha-(1→2)-β-d-Glc]	[52]
methanol	UHPLC-MS/MS	naringin, hesperidin, neohesperidin, eriodictyol, naringenin, kaempferol, hesperetin	[53]
**berries of *R. aculeatus***			
0.1% HCl in methanol	HPLC-MS	pelargonidin derivatives: 3-Glc; 3-rutinoside; 3-*p*-coumaryl-Glc	[41]
**Leaves/stems of *R. aculeatus***			
methanol	HPLC	*p*-coumaric acid, quercetin	[40]
80% methanol	HPLC-MS	caffeic acid hexoside (hex); 6 isomers of apigenin-*C*-hex.-*C*-pentoside; quercetin-*O*-deoxyhex.-hex.; kaempferol-*O*-deoxyhex.-hex.	[38]
40% ethanol	HPLC	quercetin-3-*O*-rutinoside, kaempferol-3-*O*-rutinoside, after hydrolysis: kaempferol, quercetin, *p*-coumaric acid, salicylic acid	[39]

*Glc*—*glucopyranosyl*; *Rha*—*rhamnopyranosyl*; *Gal*—*galactopyranosyl*; *Ara*—*arabinopyranosyl*; *Pen*—pentanoyl; HPLC—high-performance liquid chromatography; UHPLC—ultra-high-performance liquid chromatography; NMR—nuclear magnetic resonance; MS—mass spectrometry; ESI-MS—electrospray ionization mass spectrometry; FABMS—fast atom bombardment mass spectrometry; TLC—thin-layer chromatography; UV—ultraviolet detection; IR—infrared spectroscopy; DCCC—droplet counter current chromatography.

**Table 2:** Pharmacological effects of *R. aculeatus* on venous and lymphatic vessels.

Activity	Model	Observed Effect/Mechanism of Action	Ref.
contractile effect on venous vessels	in vitro: rings from canine saphenous veins and varicose, saphenous veins from patients in vivo (oral route): rings of saphenous veins from ovariectomized female rabbits	concentration-dependent contractile effect, activation of postjunctional α_1_- and α_2_-adrenergic receptors, stimulation of norepinephrine release	[64,65,66,67,68]
contractile effect on venous vessels	in vivo (injection): cheek pouch hamster model	constriction of venules, no effect on arterioles	[70]
contractile effect on venous vessels	in vivo (topically): cheek pouch hamster model	25 °C: arterioles and venules dilated, 36.5 °C: arterioles unchanged, venules constricted, 40 °C: arterioles unchanged or constricted, venules constricted	
venotonic effects	in vitro: veins from patients with varicose veins	increased intracellular cAMP levels, no effect on cGMP, normalization of the prostacyclin/thromboxane ratio	[71]
contractile effect on lymphatic vessels	in vitro: thoracic lymphatic ducts from dogs	Concentration-dependent contraction of the lymphatic rings; activation of adrenergic receptors	[72]
anti-edema	in vivo (topical application): cheek pouch hamster model	inhibition of microvascular permeability induced by histamine	[69]
anti-edema	in vivo (oral route): feline model	decrease in edema, reduced protein content in edema fluid, slower water flow into tissues, inhibition of endothelial destruction induced by ethacrynic acid	[73]
anti-edema/effect on endothelium	in vivo (oral route): cheek pouch hamster model	inhibition of microvascular permeability, inhibition of leukocyte-endothelium interaction (decreased adherent and rolling leukocytes)	[74]
effect on endothelium	human umbilical vein endothelial cells incubated in hypoxia conditions	decrease in ATP content, the activation of phospholipase A_2_, increase in neutrophil adhesion	[75]

**Table 5:** Cytotoxic activity of extracts from *R. aculeatus*.

Plant Part/Extract	Test (Control)	Ref.
Underground part/ 80% ethanol (e) infusions (i) decoctions (d)	HeLa GI_50_ (µg/mL): 98 (e), 320 (i), 111 (d) (ellipticine: 0.9) NCI-H460 GI_50_ (µg/mL): 51 (e), 201 (i), 69 (d) (ellipticine: 1.03) MCF-7 GI_50_ (µg/mL): 89 (e), 350 (i), 94 (d) (ellipticine: 1.21) HepG2 GI_50_ (µg/mL): 71 (e), 300 (i), 168 (d) (ellipticine: 1.10) Control line PLP2 GI_50_ (µg/mL): 179 (e), >400 (i), 265 (d) (ellipticine: 2.3)	[38]
Aerial part/ 80% ethanol (e) infusions (i) decoctions (d)	HeLa GI_50_ (µg/mL): 31 (e), 373 (i), 270 (d) (ellipticine: 0.9) NCI-H460 GI_50_ (µg/mL): 70 (e), 273 (i), 302 (d) (ellipticine: 1.03) MCF-7 GI_50_ (µg/mL): 70 (e), >400 (i, d) (ellipticine: 1.21) HepG2 GI_50_ (µg/mL): 72 (e), >400 (i), 260 (d) (ellipticine: 1.10) Control line PLP2 GI_50_ (µg/mL): 152 (e), >400 (i, d) (ellipticine: 2.3)	[38]
Aerial part/ 80% ethanol (e)	Jurkat cell IC_50_ (mg/mL): 10–16 at 24 h, 8–10 at 48 h (LDH); 5 (MTT) Control line: lymphocytes—similar results	[87]
Aerial part/water	T24 (% inhibition for 4 mg/mL): 14 (4 h), 8 (24 h), 28 (48 h), 38 (72 h) * A549 (% inhibition for 4 mg/mL): 13 (4 h), 7 (24 h), 27 (48 h), 35 (72 h) * Control line HEK 293: 35 (4 h), 32 (24 h), 18 (48 h), 0 (72 h) *	[88]

GI_50_—sample concentration responsible for 50% inhibition of growth in tumor cells; HeLa—cervical carcinoma; NCI-H460—non-small cell lung cancer; MCF-7—breast adenocarcinoma; HepG2—hepatocellular carcinoma; PLP2—primary porcine liver cells; Jurkat cell—human acute T-cell lymphoblastic leukemia cells; T24—human bladder cancer; A549—lung cancer; HEK 293—human embryonic kidney cell line; LDH—lactate dehydrogenase. *—The values were approximated from the graphs.

## Data Availability

Data is contained within the article.
